# Brain Connectivity Changes after Osteopathic Manipulative Treatment: A Randomized Manual Placebo-Controlled Trial

**DOI:** 10.3390/brainsci10120969

**Published:** 2020-12-11

**Authors:** Marco Tramontano, Francesco Cerritelli, Federica Piras, Barbara Spanò, Federica Tamburella, Fabrizio Piras, Carlo Caltagirone, Tommaso Gili

**Affiliations:** 1 IRCCS Fondazione Santa Lucia, 00174 Rome, Italy; m.tramontano@hsantalucia.it (M.T.); pirasfphd@gmail.com (F.P.); b.spano@hsantalucia.it (B.S.); f.piras@hsantalucia.it (F.P.); c.caltagirone@hsantalucia.it (C.C.); 2 Clinical-Based Human Research Department, Foundation COME Collaboration, 65121 Pescara, Italy; francesco.cerritelli@gmail.com; 3 Networks Unit, IMT School for Advanced Studies Lucca, 55100 Lucca, Italy; tommaso.gili@imtlucca.it

**Keywords:** fMRI, functional connectivity, osteopathic manipulative treatment, placebo, somatic dysfunction

## Abstract

The effects of osteopathic manipulative treatment (OMT) on functional brain connectivity in healthy adults is missing in the literature. To make up for this lack, we applied advanced network analysis methods to analyze resting state functional magnetic resonance imaging (fMRI) data, after OMT and Placebo treatment (P) in 30 healthy asymptomatic young participants randomized into OMT and placebo groups (OMTg; Pg). fMRI brain activity measures, performed before (T0), immediately after (T1) and three days after (T2) OMT or P were used for inferring treatment effects on brain circuit functional organization. Repeated measures ANOVA and post-hoc analysis demonstrated that Right Precentral Gyrus (F (2, 32) = 5.995, *p* < 0.005) was more influential over the information flow immediately after the OMT, while decreased betweenness centrality in Left Caudate (F (2, 32) = 6.496, *p* < 0.005) was observable three days after. Clustering coefficient showed a distinct time-point and group effect. At T1, reduced neighborhood connectivity was observed after OMT in the Left Amygdala (L-Amyg) (F (2, 32) = 7.269, *p* < 0.005) and Left Middle Temporal Gyrus (F (2, 32) = 6.452, *p* < 0.005), whereas at T2 the L-Amyg and Vermis-III (F (2, 32) = 6.772, *p* < 0.005) increased functional interactions. Data demonstrated functional connectivity re-arrangement after OMT.

## 1. Introduction

Osteopathic Manipulative Treatment (OMT) is a whole-body intervention mainly focused on correcting the somatic dysfunctions [1,2,3] present in different regions of the body. Osteopathic research to date has mostly been concerned with various clinical conditions such as musculoskeletal disorders [4,5], and primary headache [6,7,8]. The main advantage for patients is the effective relief of acute and chronic pain [9,10]. However, osteopathic patients are receiving osteopathic care and reporting improvements for other non-musculoskeletal complaints [11,12]. Indeed, OMT was proved effective on conditions and disorders beyond the sensory and motor system, including the reduction of hospitalization length in a large population of preterm infants [13,14], effects on anxiety and fatigue in people with multiple sclerosis [15], and on autonomic and neuroendocrine responses [16]. The neurophysiological effects underlying clinical improvements are still under debate.

Although models explaining the therapeutic effects of OMT include potential brain mechanisms, few studies have been carried out to investigate brain mechanism changes after OMT.

Magnetic resonance imaging (MRI) research includes several different approaches to estimate cortical functions. Several of these approaches have demonstrated functional brain changes associated with OMT. Using Arterial Spin Labeling MRI, we recently demonstrated that the treatment of somatic dysfunctions induces cerebral perfusion changes in asymptomatic young participants [17]. 

Cerritelli et al. [18], using resting state functional connectivity (fMRI), demonstrated that OMT induces lasting blood oxygen level-dependent (BOLD) effects on crucial areas of interoceptive networks in patients with low back pain. Further research showed that other manual therapies (namely chiropractic spinal manipulation, spinal mobilization, and therapeutic touch) have an immediate effect on functional brain connectivity [19,20]. These results suggest that the set of modifications occurring in the brain functional circuit organization after OMT can be considered an effective proxy of the neurophysiological changes following manipulative treatments. 

Low-frequency fluctuations of the fMRI signal recorded at rest are recognized to be physiologically relevant. Such spontaneous brain activity has been shown to influence behavior [21,22] as well as to contribute to variability in task-evoked responses [23,24]. Multivariate analysis of resting-state fMRI revealed the existence of networks that span across several cortical areas, consistently with the function associated with them [25,26].

Complex network theory [27] represents the state-of-the-art of multivariate analysis of local cortical and subcortical interactions [28]. Specifically, the complex network description of the functional organization of the brain demonstrates a repertoire of unexpected properties of brain connectedness [27]. Network theory allows a description of brain functioning in terms of patterns of communication among brain regions, treated as evolving networks, whose evolution is associated with behavioral outcomes [29]. Indeed, brain function is not merely attributable to individual regions and connections, as it emerges from the organization of the brain network as a whole. While regional segregation allows specialization of function, the strong integration is necessary for a number of processing operations [30]. This means that functional connections allow neural elements to orchestrate their activity into coherent dynamic states that underlie cognition and behavior. The investigation of brain networks has emerged as an exciting method to identify neural anatomical substrates of cognition and behavior. Graph modelling has become a successful approach in brain connectivity investigation. Several descriptive measures of complex networks were introduced as to identify cortical nodes that are likely to be highly influential over the behavior of the network (e.g., betweenness centrality) and in the mainstream of information flow (i.e., clustering coefficient) and to explore topological features of brain networks. 

The aim of this study was to assess the impact of OMT on cerebral functional connectivity, as derived by complex network analysis of resting-state fMRI data recorded in asymptomatic young volunteers with somatic dysfunctions. Given the differential impact exerted by OMT on cerebral perfusion [17], we hypothesize this neurophysiological effect to be reflected in OMT induced changes in network topology and organization as quantified by centrality and segregation measures, in areas involved in the dynamic balance between the sympathetic and parasympathetic systems.

## 2. Methods

### 2.1. Ethical Issues

This randomized-controlled single blinded study was approved by the Fondazione Santa Lucia’s local ethics committee with protocol number CE/PROG.625 and was conducted in accordance with the Declaration of Helsinki. Recruitment started only after the approval of the ethics committee. Clinical Trial registration number is NCT04387006.

### 2.2. Subjects

All interventions were performed at the outpatient clinic of Fondazione Santa Lucia (Scientific Institute for Research and Health Care) from September 2017 to June 2018. Participants were recruited at the Tor Vergata University of Rome. The recruitment document explained that participation was voluntary, without incentives for participants and dependent on the inclusion and exclusion criteria. All interested participants received information about the project by telephone and were briefly interviewed by a clinician not involved in the intervention sessions, to assess eligibility according to the inclusion and exclusion criteria (see below). Before participating, volunteers provided written informed consent. Forty-four asymptomatic, non-smoker, osteopathically naïve volunteers were recruited. No subject had been under any pharmacological treatment during the previous 4 weeks or had suffered from pain within the six months before the enrolment.

The inclusion criteria were: age between 18 and 40 years and suitability for MRI scanning. Exclusion criteria included: (i) cognitive impairment, based on Mini Mental State Examination (MMSE) [31] score ≤ 24 according to norms for the Italian population [32], and confirmed by a deeper clinical neuropsychological evaluation using the Mental Deterioration Battery [33] and NINCDS-ADRDA criteria for dementia [34]; (ii) subjective complaints of memory difficulties or of any other cognitive deficit, interfering or not with daily living activities; (iii) major medical illnesses, e.g., diabetes (not stabilized), obstructive pulmonary disease, or asthma; hematologic and oncologic disorders; pernicious anemia; clinically significant and unstable active gastrointestinal, renal, hepatic, endocrine, or cardiovascular system diseases; newly treated hypothyroidism; (iv) current or reported psychiatric (assessed by the Structured Clinical Interview for DSM IV Axis II Disorders (SCID-II) [35]) or neurological (assessed by a clinical neurological evaluation) disorders (e.g., schizophrenia, mood disorders, anxiety disorders, stroke, Parkinson’s disease, seizure disorder, head injury with loss of consciousness, and any other significant mental or neurological disorder); (v) known or suspected history of alcoholism or drug dependence and abuse during lifetime; (vi) MRI evidence of focal parenchymal abnormalities or cerebrovascular diseases: for each participant, a trained neuroradiologist and a neuropsychologist expert in neuroimaging co-inspected all the available clinical MRI sequences (i.e., T1- and T2-weighted and Fluid-attenuated inversion recovery (FLAIR) images) to ensure that participants were free from structural brain pathology and vascular lesions (i.e., FLAIR or T2-weighted hyper-intensities and T1-weighted hypo-intensities). Participants were asked to avoid the use of contraceptive drugs, alcohol, nicotine or other substance abuse during the study. Before the fMRI scan, patients were asked to complete paper-based questionnaires.

### 2.3. Experimental Design

This study aimed to explore the neural correlates associated with the effect of OMT in terms of cerebral functional connectivity, as derived by complex network analysis of resting state fMRI data recorded in asymptomatic young volunteers with somatic dysfunctions.

Participants were randomly divided into OMTg and the Pg through a block randomization performed according to a computer-generated pseudo-randomized list. Participants were unaware of the study design and outcome, as well as of group allocation. A researcher, not involved in the intervention sessions, performed the randomization and was the only responsible for the process and securely stored the randomization list.

**Figure 1:**
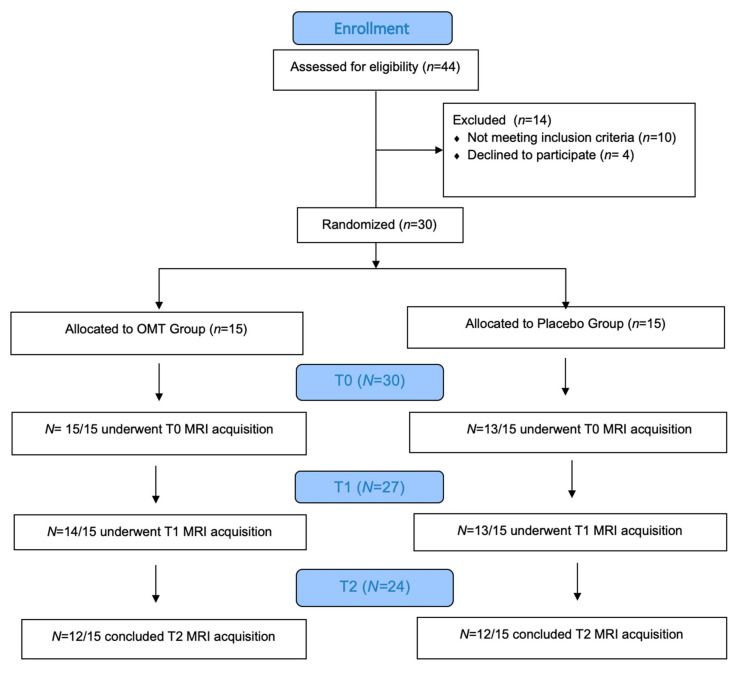
Flow Chart of the study.

All participants underwent an fMRI session before (T0), immediately after (T1) and three days after (T2) the treatment. Between T0 and T1 each participant received a single session of 45 min of OMT or P manual treatment (Figure 1).

The OMT session was performed by two female healthcare professionals who had completed a training program in osteopathy aligned with Italian Core Competencies in osteopathy [36] and with the European Standard on Osteopathic Healthcare Provision.

Somatic dysfunctions were addressed according to tissue alteration, asymmetry, range of motion and tenderness parameters (TART) which guided the osteopathic evaluation and intervention [37]. Somatic dysfunctions were detected in the whole body, then balanced one by one to define a primary order of treatment according to TART parameters. For each participant, osteopaths used the outpatient osteopathic SOAP (Subjective, Objective, Assessment, Plan) note form. OMT techniques were focused on correcting the dysfunctions found during the initial physical examination and included articular and myofascial techniques, balanced ligamentous tension, visceral manipulations and osteopathy in the cranial field [36,38,39,40]. P treatment was performed by the same osteopaths who performed OMT and was carried out in the same hospital setting. P treatment consisted of a passive touch without joint mobilization in a protocolled order [17,41]. The osteopaths were standing next to the bed, and touched the lumbar and dorsal spine of the subjects in a prone position for 10 min, then in supine position, then touched for 5 min the shoulders and the hips, for 5 min the upper and lower limbs and finally the neck, the sternum and the chest were touched for 5 min each. Another researcher expert on the placebo protocol trained the osteopaths.

### 2.4. De-Blinding Questionnaire

The de-blinding questionnaire was administered exclusively by a treatment-blinded external trained psychologist not involved in the intervention. The questionnaire consisted of three consecutive questions about subjects’ perception of the treatment received. After being questioned on whether, according to their perception, they thought they had received OMT or P treatment, subjects were asked on a 0–10 numeric rating scale (NRS), where 0 represented absolutely uncertainty and 10 represented absolutely certainty, how certain they were regarding group allocation. Finally, they were asked to rate the perceived usefulness of the treatment received, based on a 0–10 NRS, where 0 represented absolutely useless and 10 represented absolutely useful [42,43].

### 2.5. fMRI Data Acquisition

fMRI data were collected using gradient-echo echo-planar imaging at 3T (Philips Achieva) using a (T2*)-weighted imaging sequence sensitive to blood oxygen level-dependent (BOLD) (TR = 3 s, TE = 30 ms, matrix = 80 × 80, FOV = 224 × 224, slice thickness = 3 mm, flip angle = 90°, 50 slices, 240 vol). A thirty-two channel receive-only head coil was used. A high-resolution T1-weighted whole-brain structural scan was also acquired (1 mm × 1 mm × 1 mm voxels). Subjects were instructed to lay in the scanner at rest with eyes open. For the purposes of accounting for physiological variance in the time-series data, cardiac and respiratory cycles were recorded using the scanner’s built-in photo-plethysmograph and a pneumatic chest belt, respectively.

### 2.6. fMRI Preprocessing

Several sources of physiological variance were removed from each individual subject’s time-series fMRI data. For each subject, physiological noise correction consisted of removal of time-locked cardiac and respiratory artifacts (two cardiac harmonics and two respiratory harmonics plus four interaction terms) using linear regression [44], and of low-frequency respiratory and heart rate effects [45,46,47].

fMRI data were then preprocessed as follows: correction for head motion and slice-timing and removal of non-brain voxels (performed using FMRIB’s Software Library (FSL) (Analysis Group, Oxford, UK)). The first five volumes were discarded in order to stabilize the magnetization. Head motion estimation parameters were used to derive the frame-wise displacement (FWD), that in turn was used, together with its derivative, to correct data by a regression process. Timeseries were then demeaned, detrended, de-spiked and band-pass filtered in the frequency range 0.01–0.15 Hz, using custom software written in MATLAB (The MathWorks). For group analysis, a two-step registration process was performed. fMRI data were transformed first from functional space to individual subjects’ structural space using FLIRT (FMRIB’s Linear Registration Tool) and then non-linearly to a standard space (Montreal Neurological Institute MNI152 standard map) using Advanced Normalization Tools (ANTs; Penn Image Computing & Science Lab, http://www.picsl.upenn.edu/ANTS/). Finally, data were spatially smoothed (5 × 5 × 5 mm full-width half-maximum Gaussian kernel). 

### 2.7. Network Analysis

Resting state fMRI time series were averaged, for each participant, within 116 regions of interest (ROIs) of the Automated Anatomical Labeling (AAL) atlas [48]. A functional connection between two brain regions was assumed as an undirected and weighted graph link with the weighting being the square of the correlation coefficient [49]. Here the squared correlation coefficients were considered as similarity indices [50], in order to account for the sign of correlations that result from neural-mediated, temporally and spatially heterogeneous, hemodynamic mechanisms. The resulting matrix was thresholded by maintaining the graph as globally connected, i.e., implying that the number of connected elements of the graph is equal to the graph size [51]. In order to investigate the topology of the functional networks some network metrics were calculated: the eigenvector centrality [52], the degree centrality [53], the betweenness centrality [53] and the clustering coefficient [54]. 

In order to find regions where the network measures had changed due to the intervention, we calculated a repeated measures ANOVA with two levels of treatment, OMT and P, and three levels of time (T0, T1, T2), followed by post-hoc test. This analysis modelled the interaction of the effect of treatment, namely baseline pre-treatment (B) and end of treatment (E), and the effect of condition, namely OS (OMT stimulation) and PS (placebo stimulation). The interaction is described by the contrast (EOS-BOS)–(EPS-BPS) and represents OMT effects controlled by baseline scans with respect to the P. Differences were considered statistically significant at values of *p* < 0.05 FDR corrected. Data analysis was performed by means of MATLAB (The MathWorks).

We also calculated the correlation between the number of dysfunction and functional connectivity changes both for the contrast (OMT_T1–OMT_T0)–(P_T1–P_T0) and for (OMT_T2–OMT_T1)–(P_T2–P_T1). Statistical analysis was also performed for the de-blinding questionnaire, per the Mann-Whitney non-parametric test for independent samples (OMTg and Pg). Statistical significance was considered at *p* < 0.05 and the test was performed by using the software Statistical Package for the Social Science—SPSS (Chicago, IL, USA).

### 2.8. Power Analysis

To determine a sufficient sample size for two sample t-tests, power analysis was conducted using G*Power [39] based on a previous paper by Gay et al. [19], where three different types of manual therapies determined FC changes immediately after the treatment.

Using alpha equal to 0.05, power equal to 0.80 and Cohen’s d equal to 0.55 (the OMT in our study was not restricted to osteopathy in the cranial field) the desired sample size for the difference between two dependent means was 22 subjects in total.

## 3. Results

### 3.1. Description of the Sample at Baseline

44 asymptomatic young volunteers were evaluated for eligibility, as reported below in the “Methods–Subjects” section. Given the inclusion/exclusion criteria, 30 out of 44 participants were enrolled in this trial. These participants were randomized into OMT or Placebo groups, correspondingly, OMTg (*n* = 15) and Pg (*n* = 15). Information about study design is reported in the study flow chart (Figure 1).

Before the treatment (baseline or T0), directly after (T1), and 3 days later (T2) the participants underwent an fMRI session. After T0 and before T1 all participants underwent a single 45 min session of OMT or P treatment. Later, participants were asked to fulfill a de-blinding questionnaire. This questionnaire was composed of three questions about participant’s expectations of treatment as detailed in [17]. 

Demographic features of participants are reported in Table 1.

**Figure 2:**
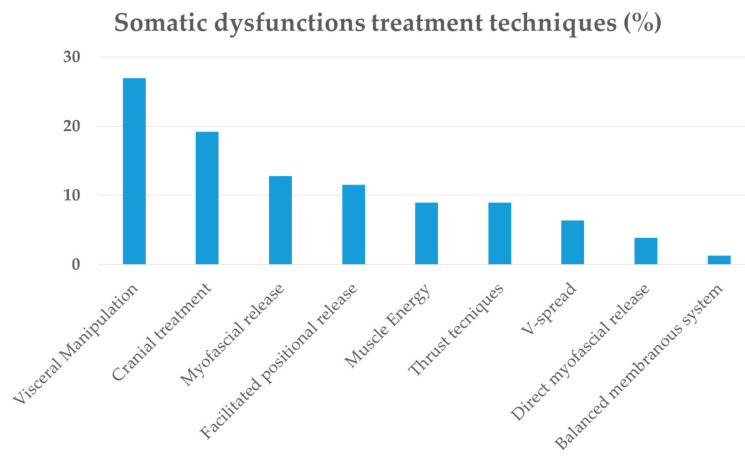
Somatic dysfunction treatment techniques (%).

Statistical assessment did not show significant differences between groups as regards years of education, age and sex (*p* > 0.05). For statistical details, see section “Methods-Network analysis”. For OMTg, details of the localization of treated dysfunctions with a report of the different techniques used are reported in Table 2 and Figure 2, respectively.

### 3.2. fMRI Results

Two participants from the Pg were excluded because of referred pain perception during T0 MRI acquisition, while one participant from the OMTg dropped out at T1 due to a later developed MRI intolerance. At T2, two OMTg participants dropped out for a sudden MRI intolerance, and one Pg participant dropped out for personal reasons. Details about OMT or P treatment received by subjects are reported in the section “Methods-Experimental design”. 

The repeated measures ANOVA showed that mean betweenness centrality of Right Precentral Gyrus (R-PrecentralG; F(2, 32) = 5.995, *p* < 0.005) and Left Caudate (L-Cau; F(2, 32) = 6.496, *p* < 0.005), and mean clustering coefficient of Left Middle Temporal Gyrus (L-MTG; F(2, 32) = 6.452, *p* < 0.005), Left Amygdala (L-Amyg; F(2, 32) = 7.269, *p* < 0.005) and the lobule three of the cerebellar vermis (Vermis-III; F(2, 32) = 6.772, *p* < 0.005) differed significantly between time points, once the interaction of the effect of “dosing” the treatment, namely baseline pre-treatment (T0), post-treatment (T1) and follow-up (T2), and the effect of “administering” the treatment, namely P or OMT, was investigated. 

This analysis modelled the interaction of treatment effect (namely baseline pre-treatment (B) and end of treatment (E)), and the effect of condition (namely OS (OMT stimulation) and PS (P stimulation)). The interaction is described by the contrast (EOS-BOS)–(EPS-BPS) and represents OMT effects controlled by baseline scans with respect to P. The post-hoc (EOS-BOS)–(EPS-BPS) contrast showed significant pre/post treatment and post-treatment/follow-up changes (*p* < 0.05).

#### 3.2.1. Pre-Treatment to Post-Treatment Changes (T0 vs. T1 fMRI Data)

**Figure 3:**
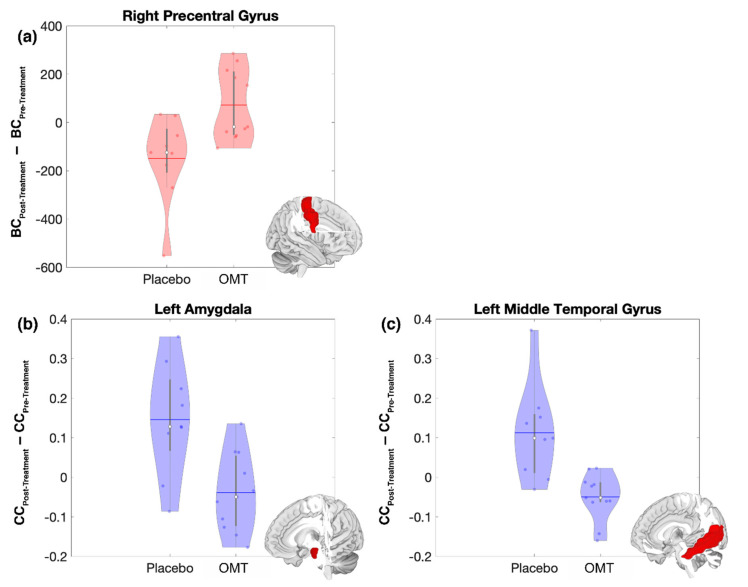
Pre to Post-treatment changes of brain functional topological measures in the Right Precentral Gyrus (**a**), Left Amygdala (**b**) and in the Left Middle Temporal Gyrus (**c**). Violin plots of the statistically significant difference between the post-treatment and pre-treatment values of Betweenness Centrality (BC, red) and Clustering Coefficient (CC, blue) for the Osteopathic Manipulative Treatment (OMT) and Placebo (P). The solid horizontal lines represent the mean of the distribution, while the white dots represent the median. For each plot the structural representation of the associated region is reported as the red portion of the whole brain (right Precentral Gyrus, left Amygdala and left Middle Temporal Gyrus respectively).

An increase in betweenness centrality, as an effect of OMT, was found in the R-PrecentralG, as opposed to a decrease after P treatment (Figure 3a). Similarly, a decreased clustering coefficient was found both in the L-Amyg and in the L-MTG, due to OMT, while an increase was found after P treatment (Figure 3b,c).

#### 3.2.2. Post-Treatment to Follow-Up Changes (T1 vs. T2 fMRI Data)

**Figure 4:**
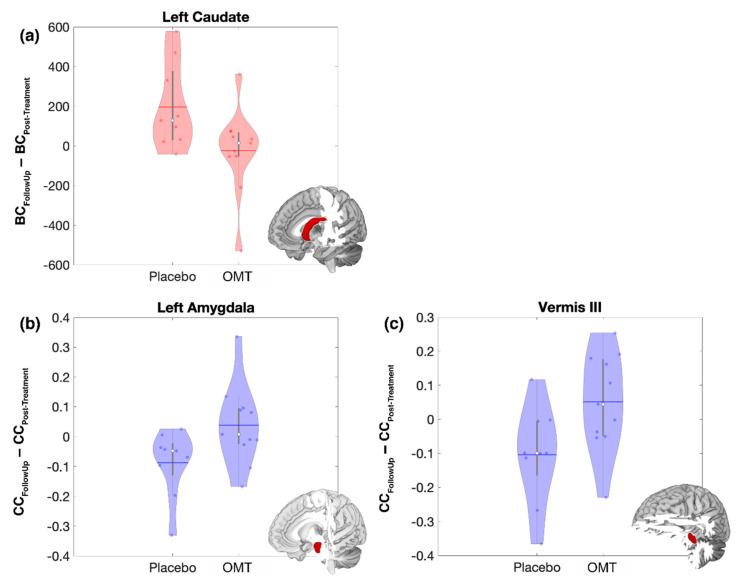
Post-treatment to Follow-Up changes of brain functional topological measures in the Left Caudate (**a**), Left Amygdala (**b**) and in the Vermis III (**c**). Violin plots of the statistically significant difference between the follow-up and post-treatment values of Betweenness Centrality (BC, red) and Clustering Coefficient (CC, blue) for the Osteopathic Manipulative Treatment (OMT) and Placebo (P). The solid horizontal lines represent the mean of the distribution, while the white dots represent the median. For each plot the structural representation of the associated region is reported as the red portion of the whole brain (left Caudate, Left Amygdala and Vermis III respectively).

A decrease in betweenness centrality, as an effect of OMT, was found in the L-Cau, while the same area showed an increase after P treatment (Figure 4a). Along with that, an increase in clustering coefficient was found both in the L-Amyg and in the Vermis-III, due to OMT, as opposed to a decrease after P treatment (Figure 4b,c).

### 3.3. De-Blinding Questionnaire Results

Regarding the de-blinding questionnaire, 75% of the OMTg and 41.6% of the Pg answered that they underwent OMT. Ratings mean of both groups regarding treatment allocation certainty was 7.0 ± 1.6 and ratings means of treatment usefulness was 7.5 ± 2.6 for OMTg and 6.6 ± 1.9 for Pg. No statistical difference between OMTg and Pg was observable regarding subjects’ assumption, certainty or perceived usefulness of received treatment. 

Furthermore, concerning the correlation between the number of dysfunction and functional connectivity changes both for the contrast (OMT_T1–OMT_T0)–(P_T1–P_T0) and for (OMT_T2–OMT_T1)–(P_T2–P_T1), no significant correlation emerged (*p* > 0.05). No adverse effects were reported for any participant.

## 4. Discussion

The study aimed to investigate the effects of OMT on cerebral functional connectivity compared to P treatment in healthy asymptomatic young adults with somatic dysfunctions. Results showed significant changes between OMTg and Pg in two centrality measures expressing functional segregation at the local level, and the influence each region had on the information flow (betweenness centrality and clustering coefficient) for the comparisons T0 vs. T1 and T2 vs. T1, but not between T0 vs. T2, suggesting reversible treatment effects. 

Besides, data obtained showed opposite effects between OMTg and Pg. Indeed, betweenness centrality in the OMTg increased in the R-PrecentralG, and decreased in the L-Cau. This between-groups opposite behavior was also observable for clustering coefficient. These results may suggest that an immediate OMT effect is the strengthened connectedness and increased segregation in the R-PrecentralG, while a delayed OMT effect is the reduction of L-Cau functional segregation with a consequent enhancement of information flow within this region. Moreover, L-Amyg was the only brain area that consistently responded to treatment, displaying a marginal decrease in clustering coefficient immediately after OMT (T1) and a marginal increase after 3 days (T2). Thus, the immediate OMT effect was not mediated by functional segregation within the amygdala, which became more influential over the information flow in the delayed OMT effect.

Interestingly, in order to discuss further this result, it was reported that amygdala receives inputs from sensory systems arising from association areas [55]. 

These, supply the amygdala with a more elaborate representation which could be provided by the thalamic inputs. Moreover, the involvement of additional synaptic connections makes the transmission slower [55]. Besides, amygdala central nucleus output is directed to the dorsal nucleus of Vagus nerve [55]. Data of our previous study [17] showed, immediately after OMT, a posterior cingulate cortex (PCC) perfusion reduction related to a predominance of sympathetic modulation, while after 3 days, a change in the direction of a moderately larger vagal predominance was observed in terms of PCC perfusion increment. 

It is significant, for the present evidence, to consider the PCC as a critical node of the central autonomic network [56] which controls preganglionic sympathetic and parasympathetic motoneurons, having an important involvement in the parasympathetic functioning control [56,57,58]. 

These results underlined, therefore, a possible modulation of the autonomic system as an effect of OMT as reported also in previous studies [59,60]. Thus, the early reduction in amygdala neighborhood connectivity and the successive reversed pattern may explain the initially sympathetic predominance followed by a relatively larger vagal predominance. Amygdala has also a role in the reward processing and in the using of the reward in order to reinforce and motivate the behavior [55]. No statistical difference between groups were pointed out as concerns subjective perception of the received treatment, even if the perceived usefulness of OMT was higher than P treatment (*p* > 0.05). We can speculate that OMT, considered as more useful than P treatment, can increase subjects’ motivation, thus considering treatment as a reward. 

In line with this data, the amygdala is also devoted to identifying and effectively learning important events in the environment that are emotionally and motivationally relevant [61]. It is plausible that subjects who underwent OMT perceived stimuli as emotionally and motivationally relevant after 3 days of integration time [17].

Furthermore, an increased motor cortical excitability in subjects who had undergone osteopathic treatment was shown in a study carried out with transcranial magnetic stimulation [62]. These results, therefore, indicate specific changes in brain connectivity which involve not only the nociceptive/emotional/autonomic network, as predictable, but also sensorimotor, locomotor and postural network, which includes the prefrontal cortex, basal ganglia, brainstem, and cerebellar locomotor centers. This suggest a change in the information processing occurred after the treatment and agrees with a previous study investigating functional connectivity using resting-state functional MRI [18]. 

The analyzed data of the resting-state fMRI of patients with low back pain revealed several brain resting-state network fluctuations such as in the interoceptive/autonomic network compared to sham controls [18].

Furthermore, closely related studies have recently been reported to investigate brain signatures in connectivity or source signals for outcome prediction using a placebo-controlled clinical trial [63,64,65].

Interestingly, our results showed that information flow in the L-Cau and functional segregation in the Vermis-III significantly increased for subjects who underwent OMT at T2 in comparison to T1 suggesting later onset modifications. Basal ganglia are group of subcortical nuclei able to integrate information from cortical areas to the cerebral cortex. The basal ganglia system is also discussed by Caudate [66] and is involved in “the automatic or unconscious control of movements that accompany voluntary movements, such as rhythmic limb movements and adjustment of postural muscle tone during locomotion, which occurs in conjunction with voluntary control processes” [66]. The freedom level of the automatic and volitional aspects of movements are respectively defined by the basal ganglia outputs toward the brainstem and the thalamocortical loop [66]. Based on these premises, we can hypothesize that OMT may induce a modulation of subcortical structures, consequently permitting an adjustment in muscle tone to better manage postural control and programming of movements. Besides, the information provided by the different OMT techniques used here might have stimulated the sensorimotor area with consequent excitatory effects on the striatal cells. In asymptomatic individuals the presence of somatic dysfunctions generates biomechanical and neurological repercussions, especially tissue texture changes and nociceptors activation with a consequent arising of tissue inflammation [67]. Given this, we might hypothesize that somatic dysfunctions treatment may generate central reversible changes [68,69], consequently generating the reported less functional segregation in the basal ganglia. The particular nature of basal ganglia contribution to normal somatosensory perception is undefined. In case of basal ganglia lesion or dysfunction, the conscious pressure or touch perception are not lost; nonetheless, sensory and cognitive functions of basal ganglia activity can disturb features of cutaneous and kinesthetic discernment as well as somatosensory feedback for movement [70]. Furthermore, the caudate nucleus has been shown to be activated during a simulation of stress [71]. It is possible that re-organization induced by OMT may be perceived by participants as an external stressor factor, with a consequent increase of caudate connectivity. All these effects on L-Cau were reversible because no difference in T0 vs T2 comparison were observed for L-Cau connectivity.

In symptomatic participants (i.e., with pain) more evident responses to general OMT intervention might be elicited, and further research could investigate whether these improvements correspond with beneficial clinical results, thus being interpretable as a result of somatic dysfunction correction and due to the brain connectivity changes. Although enrolling patients might be certainly appropriate, the use of healthy participants has a double-fold advantage: firstly, it can be useful to identify physiological responses which are not disease-related; secondly it can create baseline data to allow comparisons and benchmarks when future patient-based research is performed.

## 5. Conclusions

According to our knowledge, no previous study has analyzed OMT vs P treatment effects on brain functional connectivity per the fMRI. Therefore the present study aimed to analyze and compare brain functional connectivity before, immediately after, and 3 days after OMT and P treatments, and showed a distinct functional and reversible connectivity re-arrangement of the supraspinal locomotor network, consisting of the premotor cortex, basal ganglia, and midline cerebellum, as well as the emotional/autonomic network, with specific activation of the amygdala.

This research provides the first preliminary evidence of brain network connectivity changes due to OMT, opening further insights into potential effects of OMT on brain functional activity. Moreover, it suggests future investigations in this unexplored field, particularly on symptomatic subjects. Personal beliefs on betweenness centrality and clustering coefficients, considering amygdala connections and the opposite L-Amyg data obtained at T1 and T2 assessments, could also be investigated.

## Figures and Tables

**Table 1:** Participants’ demographic characteristics.

	Osteopathic Manipulative Treatment Group (OMTg) (*n* = 15)	Placebo Group (Pg) (*n* = 15)	*t*, χ^2^
Age (years) ^a^	28.0 ± 5.5	25.4 ± 3.2	^c^*t*_(28)_ = −1.6, *p* = 0.1 95% CI = −6.0, 0.8
Gender (M/F) ^b^	8/7	4/11	^d^ χ_(1)_ = 2.2, *p* = 0.3
Education (years) ^a^	16.0 ± 1.5	16.1 ± 0.4	^c^*t*_(28)_ = 0.2, *p* = 0.9 95% CI = −0.8, 0.9
Body Mass Index ^a^	20.9 ± 6.1	21.0 ± 6.6	^c^*t*_(28)_ −0.8, *p* = 0.9 95% CI = −4.6, 4.9

Value are expressed as ^a^ mean ± standard deviation or ^b^ number. Groups comparisons were performed with ^c^ independent-sample *t*-tests and ^d^ Chi-square tests.

**Table 2:** Dysfunctions localization reported for the different body segments in OMTg according to somatic dysfunction classifications.

Dysfunctions Localization	%
M99.01 Cervical	28.1
M99.0 Head	26.9
M99.09 Abdomen and other regions	22.4
M99.05 Pelvic	6.4
M99.02 Thoracic	6.2
M99.03 Lumbar	3.6
M99.08 Rib cage	2.5
M99.04 Sacral	1.3
M99.06 Lower extremity	1.3
M99.07 Upper extremity	1.3
